# Initial empirical antibiotics of non-carbapenems for ESBL-producing *E. coli* and *K. pneumoniae* bacteremia in children: a retrospective medical record review

**DOI:** 10.1186/s12879-022-07881-7

**Published:** 2022-11-21

**Authors:** Saera Park, HyeJin So, Mi-Na Kim, Jina Lee

**Affiliations:** 1 Department of Pediatrics, Asan Medical Center, University of Ulsan College of Medicine, 88, Olympic-Ro 43-Gil, Songpa-Gu, Seoul, 05505 Republic of Korea; 2 Department of Laboratory Medicine, Asan Medical Center, University of Ulsan College of Medicine, Seoul, Republic of Korea

**Keywords:** ESBL producers, Bacteremia, Empirical antibiotics, Carbapenem, Children

## Abstract

**Background:**

The efficacy of non-carbapenems as an empirical antibiotic for extended-spectrum β-lactamases (ESBL)-producing *Escherichia coli* and *Klebsiella pneumoniae* bacteremia in children remains controversial. We compared clinical and microbial outcomes according to the types of empirical antibiotics for treating pediatric patients with ESBL-producing *E. coli* and *K. pneumoniae* bacteremia.

**Methods:**

Data from pediatric patients aged ≤ 18 years who were hospitalized with monomicrobial ESBL-producing *E. coli* or *K. pneumoniae* bacteremia at Asan Medical Center Children’s Hospital, Seoul, Korea between January 2014 and May 2019 were analyzed retrospectively. The impact of empirical therapy was assessed as 30-day all-cause mortality and 2-day microbiological outcomes evaluated by the sterility of blood cultures collected on day 2 after empirical antibiotic administration. Logistic regression analysis was used to control for the effects of confounding variables.

**Results:**

A total of 53 patients with bacteremia caused by ESBL-producing *E. coli* (n = 29) and *K. pneumoniae* (n = 24) were included in this study; the median age was 3.6 years, and all had underlying comorbidities. As empirical antibiotics, 27 patients were treated with meropenem, and non-carbapenem agents were administered to 26 patients; 84.6% (22/26) were converted to carbapenem antibiotics as the definitive antibiotic by day 2 after empirical antibiotic administration. Overall, the 30-day all-cause mortality of ESBL-producing *E. coli* and *K. pneumoniae* bacteremia was 17.0% (9/53). After adjustment, there was no statistically significant association of use of a non-carbapenem agent as an empirical antibiotic with microbiological failure on day 2 and 30-day all-cause mortality [adjusted odds ratio (OR) 1.0; 95% confidence interval (CI) 0.22–4.88, and adjusted OR 0.1; 95% CI 0.01–1.56].

**Conclusions:**

The empirical use of non-carbapenems might not be a risk factor for mortality and early microbiological outcomes in pediatric patients with ESBL-producing *E. coli* and *K. pneumoniae* BSI if early transition to appropriate antimicrobial therapy was possible.

**Supplementary Information:**

The online version contains supplementary material available at 10.1186/s12879-022-07881-7.

## Introduction

*Enterobacteriaceae* are important opportunistic pathogens and common microflora, but can cause severe diseases including meningitis, pneumonia, pyelonephritis, intra-abdominal infection, and septicemia (1). Antibiotic resistant has been increased all over the world which is considered a public health threat, and the emergence of multidrug-resistant (MDR) bacterial pathogens of various origins including humans and animals, which have increased the need for screening of MDR strains to select appropriate antibiotics (2). Molecular epidemiology of MDR or extended-spectrum β-lactamases (ESBLs) can be important because the high-risk clones can disseminate and acquire more antimicrobial resistant determinants; the dominant spread of CTX-M-15 and high-risk international clones of ST131 were reported in Portugal and Spain (3, 4). However, diverse clones and ESBL types were prevalent by period and hospital in Korea (5, 6).

The global number of infections caused by antibiotic-resistant *Escherichia coli* and *Klebsiella pneumoniae* in 2014 was high: 50·1 million serious third-generation cephalosporin-resistant infections and 3·1 million serious carbapenem-resistant infections (7). One of the main mechanisms for resistance to 3rd generation cephalosporins (CSs) is the production of β-lactamases including ESBLs, which are plasmid-mediated β-lactamases that can hydrolyze most β-lactams such as penicillin, aztreonam, and extended-spectrum CSs (8).

Bloodstream infections (BSIs) caused by extended-spectrum β-lactam-resistant *E. coli* and *K. pneumoniae* are increasing public health concerns worldwide with significant morbidity and mortality in children (9–11). A meta-analysis of pediatric BSIs from 1996 to 2013 showed the prevalence of ESBL-producing *Enterobacteriaceae* (hereinafter referred to as ESBL-producers) accounted for 9% of BSIs, with an annual increase of 3.2%, and a higher prevalence of 11% was observed in neonates (12). However, the prevalence of ESBL producers among *E. coli* and *K. pneumoniae* has already been reported to be as high as 50% at a tertiary children’s hospital in Korea (13). BSIs caused by ESBL-producers are associated with poor outcomes including increased mortality and long hospital stays in adults and children (9–11). In particular, in neonates infected with ESBL-producers, the all-cause mortality is reported to be as high as 36% (12, 14–16).

Carbapenem antibiotics have been recommended as the drugs of choice for serious infections such as BSIs or pneumonia caused by ESBL-producers. However, along with the increased incidence of ESBL-producing Gram-negative infections worldwide, increased use of carbapenems has exerted selection pressure on carbapenem resistance and the spread of carbapenemases (17). As a carbapenem-sparing option for infections caused by ESBL- producers, β-lactam/β-lactamase inhibitors (BLBLIs) can be considered as an alternative option. Although ESBLs are defined as β-lactamases that are inhibited by clavulanate in vitro, there are concerns that in vitro susceptibility to β-lactamase inhibitors might not translate into clinical efficacy, especially in high inoculum infections (17). Furthermore, findings from a recent randomized clinical trial including hospitalized adult patients with ceftriaxone-resistant *E. coli* or *K. pneumoniae* BSIs did not support the use of piperacillin-tazobactam as a definitive treatment (18).

However, whether the use of carbapenem antibiotics as an initial empirical regimen is associated with better outcomes than the use of non-carbapenem antibiotics such as BLBLIs remains controversial (19). Few studies have examined the efficacy of BLBLIs as an initial empirical regimen against BSIs caused by ESBL-producers (19–21). Furthermore, relevant data on empirical regimens for ESBL producers in children are lacking.

In this study, we aimed to compare the clinical and microbiological outcomes of pediatric patients with bacteremia caused by ESBL-producing *E. coli* and *K. pneumoniae* who were given carbapenem and non-carbapenem antibiotics as initial empirical regimens. The impact of initial empirical therapy on clinical and microbiological outcomes was assessed as 30-day all-cause mortality and the sterility of blood cultures collected on day 2 after initial empirical antibiotic administration, respectively.

## Materials and methods

### Study population

This is a single-center, retrospective, and observational study comparing the outcomes of patients with ESBL-producing *E. coli* or *K. pneumoniae* bacteremia who were treated with different initial empirical antibiotics. Pediatric patients aged ≤ 18 years who were hospitalized with monomicrobial ESBL-producing *E. coli* or *K. pneumoniae* bacteremia at Asan Medical Center Children’s Hospital, Seoul, Korea between January 2014 and May 2019 were included. Clinical and demographic data were abstracted from electronic medical records including the clinical diagnosis, underlying medical conditions, antibiotics administered, prior intensive care unit (ICU) state, presentation with septic shock, need for a higher level of respiratory support, and all-cause 30-day mortality. Only the first isolate was included during a single clinical episode occurring within 4 weeks, and duplicates from the same patient were excluded. The exclusion criteria were as follows: (1) polymicrobial BSI, (2) BSI caused by *E. coli* or *K. pneumoniae* without an ESBL phenotype, (3) BSI caused by carbapenem-resistant isolates, and (4) patients who did not receive a carbapenem as the definitive therapy even though ESBL production was confirmed.

The primary focus of BSIs was classified as follows; central-line associated BSIs (laboratory-confirmed BSI in which an eligible BSI organism was identified and an eligible central line present on the date of event or the day before,CLABSI) (22), urinary tract infection (UTI), intra-abdominal infection, pneumonia, or none. Our institute performs cerebrospinal fluid (CSF) culture as well as blood culture in infants younger than 3 months of age who are suspected of having clinical sepsis before initiating empirical antibiotics. However, if the feasibility of a lumbar puncture is controversial, such as in extremely low birth weight infants or patients with unstable vital signs, empirical antibiotics may be administered immediately after obtaining blood cultures without performing a lumbar puncture; in the case of infants aged < 3 months with BSIs caused by ESBL-producing *E. coli* or *K. pneumoniae*, the dosage and duration of meropenem as a definitive therapy is adjusted on the premise that meningitis is present. As for CLABSI, it was recommended that catheters were removed from patients with suppurative thrombophlebitis, endocarditis, or persistent bacteremia despite > 72 h of antimicrobial therapy to which the infecting microbes are susceptible (23). In uncomplicated CLABSI, however, the catheter might be left in place if too difficult to replace because of the limited access sites.

### Definitions

Initial presentation with severe infection was defined as the need for a higher level of respiratory support or presentation with shock. The shock was defined as the need to use a new inotropic or to increase the dose of an inotropic to maintain blood pressure. The need for a higher level of respiratory support was defined as whether respiratory support at a higher stage was required at least 3 days before the blood culture test was performed and was classified as none (room air), low-flow system (nasal cannula, simple mask), high-flow system (high-flow nasal cannula, continuous positive airway pressure), and mechanical ventilation. Empirical therapy was defined as appropriate if started within 24 h of initial blood culture collection and if the isolate was in vitro susceptible. Definitive antibiotics referred to the antibiotic therapy that was administered after to receipt of antibiotic susceptibility test results. Microbiological failure on day 2 was defined as failure of sterility of blood cultures collected on or before calendar day 2 after empirical antibiotic administration.

### Antibiotics and outcomes

Each empirical and definitive antibiotic was administered according to the ‘*Pediatric and Neonatal Dosage Handbook*’ (24), and each dose was infused over 30 min. Although carbapenems are recommended as definitive antibiotics for invasive infections caused by ESBL-producers, some patients diagnosed with UTI ± bacteremia who show improvement in clinical symptoms and bacterial sterilization may maintain the initial non-carbapenem antibiotic as the definitive therapy. Among carbapenems, meropenem was used when meningitis was diagnosed or meningitis could not be excluded.

Clinical outcome of patients with ESBL-producing *E. coli* or *K. pneumoniae* BSI was assessed as 30-day all-cause mortality. The microbiological outcome on day 2 was evaluated by the sterility of blood cultures collected on day 2 after empirical antibiotic administration.

### Isolation, identification, and antimicrobial susceptibility test of *E. coli* and *K. pneumoniae*

Aseptically collected blood was inoculated into BACTEC PEDS PLUS/F and BACTEC LYTIC Anaerobic/F Bactec Plus (BACTEC 9240, BD, Sparks, MD, USA). Species identification and antimicrobial susceptibilities were performed using the MicroScan WalkAway 96 plus system and Neg Combo Panel Type 72 (Beckman Coulter, Brea, CA, USA). ESBL producers were determined according to the Clinical and Laboratory Standards Institute M100 (2019) (25), which measures the minimum inhibitory concentration around both cefotaxime and ceftazidime disks with or without clavulanate for *E. coli*, *K. pneumoniae*, *K. oxytoca*, and *Proteus mirabilis*.

### Statistical analysis

To compare the baseline characteristics between the two empirical treatment groups, the Mann–Whitney U test or Chi square test was used. In identifying the independent risk factors for 30-day all-cause mortality and microbiological outcome on day 2, logistic regression analysis was used to control for the effects of confounding variables. *P* values < 0.05 were considered statistically significant. Statistical analyses were performed using SPSS version 20.0 (IBM Software, Chicago, IL, USA).

### Ethical approval and consent to participate

This study was approved by the Institutional Review Board of Asan Medical Center with a waiver of informed consent due to its retrospective, de-identified data collection and analysis (IRB No. 2019-0617). All methods were carried out in accordance with relevant guidelines and regulations.

## Results

### Demographic and clinical characteristics

**Fig. 1:**
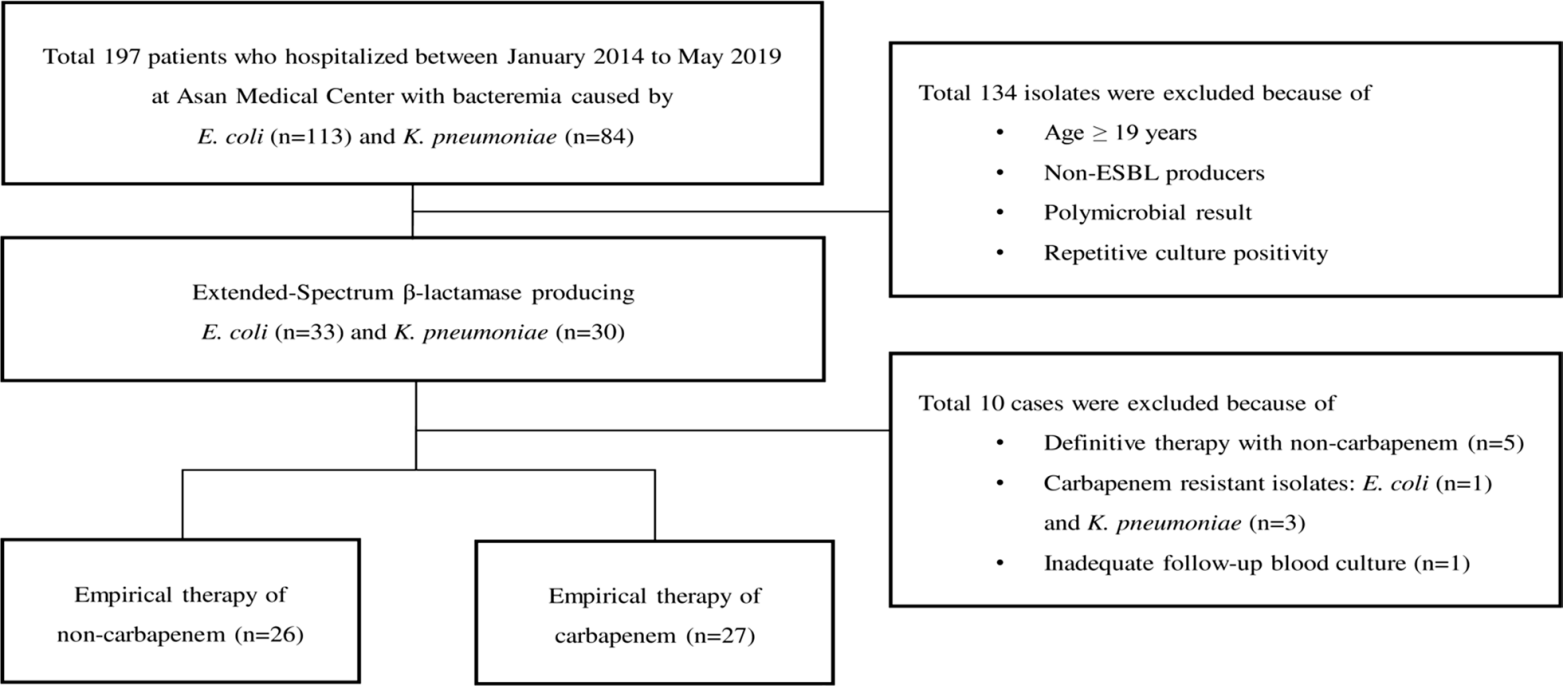
Patient inclusion flowchart

During the study period over 5 consecutive years, a total of 197 isolates of *E. coli* (n = 113) and *K. pneumoniae* (n = 84) were detected from blood cultures at Asan Medical Center Children`s Hospital (Fig. 1). A total of 134 isolates were excluded from this analysis for the following reasons: isolates obtained from the patients aged ≥ 19 years, non-ESBL producers, repetitive culture positivity, or multiple bacteria from a blood culture obtained simultaneously. In addition, 10 isolates were also excluded because 5 isolates were obtained from patients with urosepsis who showed improvement in clinical symptoms and bacterial sterilization while maintained on the initial non-carbapenems as the definitive therapy, 4 were carbapenem-resistant organisms, and one patient did not have a follow-up blood culture.

Finally, a total of 53 (26.9%) patients with BSIs caused by ESBL-producing *E. coli* (n = 29) and *K. pneumoniae* (n = 24) were included in this study. Baseline demographic and clinical data according to initial empirical therapy are presented in Table 1. The median age was 3.6 years (range 0–18.8 years), and all of the patients had underlying comorbidities such as hemato-oncologic diseases (n = 26) and prematurity with a gestational age < 37 weeks (n = 11). The most common source of bacteremia was a central vascular catheter (n = 38), followed by intra-abdominal infection (n = 6), and UTI (n = 4). Overall, 39.6% (21/53) of BSIs occurred in ICU patients, and 32.1% (17/53) of patients presented with severe infections at the onset of bacteremia including septic shock (26.4%; 14/53) and/or the requirement for higher respiratory support (22.6%; 12/53).

**Table 1:** Baseline characteristics of the study population

Characteristics	Initial empirical antibiotics			*P*-value
	Non-carbapenem (N = 26)	Carbapenem (N = 27)	Total (N = 53)	
Median age, years (range)	3.2 (0–18.7)	3.8 (0–18.8)	3.6 (0–18.8)	0.60
Sex (M:F)	17:9	14:13	31:22	0.32
Strain (*E. coli*: *K. pneumoniae*)	17:9	12:15	29:24	0.13
Presence of underlying diseases	26 (100%)	27 (100%)	53 (100%)	
Hemato-oncologic disease	13 (50%)	13 (48.1%)	26 (49.1%)	0.90
Preterm infant (GA < 37 wks)	3 (11.5%)	8 (29.6%)	11 (20.8%)	0.11
Congenital heart disease	3 (11.5%)	4 (14.8%)	7 (13.2%)	0.73
Vesicoureteral reflux	2 (7.7%)	0 (0%)	2 (3.8%)	0.14
Others	5^†^ (19.2%)	2^‡^ (7.4%)	7 (13.2%)	0.20
Primary focus of bacteremia				
None	2 (7.7%)	0 (0%)	2 (3.8%)	0.14
CLABSI	16 (61.5%)	22 (81.5%)	38 (71.7%)	0.11
Intra-abdominal infection	1 (3.8%)	5 (18.5%)	6 (11.3%)	0.09
NEC	1 (3.8%)	4 (14.8%)	5 (9.4%)	0.17
Peritonitis	0 (0%)	1 (3.7%)	1 (1.9%)	0.32
Urinary tract infection	4 (15.4%)	0 (0%)	4 (7.5%)	0.03
Pneumonia	2 (7.7%)	0 (0%)	1 (1.9%)	0.30
Meningitis	1 (3.8%)	0 (0%)	1 (1.9%)	0.30
ICU stay prior to infection	8 (30.8%)	13 (48.1%)	21 (39.6%)	0.20
Initial presentation with severe infection	4 (15.4%)	13 (48.1%)	17 (32.1%)	0.01
Presentation with shock	3 (11.5%)	11 (40.7%)	14 (26.4%)	0.02
Need for higher respiratory support	3 (11.5%)	9 (33.3%)	12 (22.6%)	0.06

*BSI* blood stream infection, *CLABSI* central-line-associated blood stream infection, *GA* gestational age, *ICU* intensive care unit, *NEC* necrotizing enterocolitis

^†^Includes ornithine transcarbamylase deficiency (n = 1), Alagille syndrome (n = 1), bronchopulmonary dysplasia (n = 1), chronic kidney disease (n = 1), and meningomyelocele (n = 1)

^‡^Includes DiGeorge syndrome (n = 1) and liver transplantation state (n = 1)

### Baseline characteristics according to initial empirical antibiotics

Overall, the initial empirical antibiotic groups were balanced with respect to the baseline characteristics, although more patients in the non-carbapenem group than in the carbapenem group had vesicoureteral reflux and a diagnosis of UTI (*P* = 0.14 and *P* = 0.03, respectively) (Table 1). More patients in the carbapenem group than in the non-carbapenem group had a history of prematurity and were diagnosed with intra-abdominal infection (*P* = 0.11 and *P* = 0.09, respectively). The incidence of initial presentation with septic shock was significantly higher in the carbapenem group than in the non-carbapenem group (41% vs 12%; *P* = 0.02).

### Initial empirical antibiotics and outcomes

As initial empirical antibiotics, 27 patients were treated with carbapenems, all of which were meropenem, and non-carbapenem agents were administered to 26 patients. Non-carbapenem antibiotics included cefepime (n = 14), cefotaxime (n = 4), piperacillin/tazobactam (n = 6), and amikacin (n = 2), all of which were applied with or without glycopeptides at the discretion of the physicians (Table 2). In the non-carbapenem group, only 23.1% (6/26) were appropriate empirical antibiotics; 84.6% (22/26) were converted to a carbapenem as the definitive antibiotic by day 2 after empirical antibiotic administration. Another 2 cases were converted by day 3, and one was converted by day 4. Among 3 patients who were maintained non-carbapenem antibiotics for 3 or 4 days after initial blood culture collection, microbiological failure on day 2 occurred in 2 patients; one was treated with an in vitro susceptible empirical antibiotic of piperacillin/tazobactam for 4 days, and the other was treated with an in vitro resistant cefepime for 3 days; however, there were no fatal cases.

**Table 2:** Types and duration of administration of non-carbapenems as initial empirical antibiotic

Non-carbapenem antibiotics	Number of cases (total n = 26)
Types	
Amikacin	2 (7.7%)
Cefepime	14 (53.8%)
Cefotaxime	4 (15.4%)
Piperacillin/Tazobactam	6 (23.1%)
Duration of administration (calendar days)	
1	16 (61.5%)
2	7 (26.9%)
3	2 (7.7%)
4	1 (3.8%)

There were a total of 9 fatal cases; 8 in the carbapenem groups and 1 in the non-carbapenem group (Additional file 1).

One patient who was on chemotherapy for underlying malignancy and experienced fatality in the non-carbapenem group was initially administered cefepime that was in vitro resistant and died within one day before switching to carbapenems. Two more fatal cases occurred in the malignancy patients with chemotherapy-induced neutropenia were initially treated with carbapenems. During our study period, an outbreak of ESBL-producing *K. pneumoniae* bacteremia among extremely low birth weight infants in the neonatal ICU (NICU) resulted in four fatal cases associated with necrotizing enterocolitis (NEC) that were all initially treated with carbapenems and died within 2 days. Furthermore, two more fatal cases were observed in preterm infants as sporadic cases.

Overall, the 30-day all-cause mortality of ESBL-producing *E. coli* and *K. pneumoniae* bacteremia was 17.0% (9/53); 29.6% (8/27) in those receiving a carbapenem antibiotic and 3.8% (1/26) in those receiving a non-carbapenem antibiotic (Table 3). Microbiological failure by day 2 occurred in 7 of 26 patients (26.9%) in the non-carbapenem group compared with 11 of 27 (40.7%) patients in the carbapenem group (*P* = 0.29).

**Table 3:** Clinical Outcomes of the Study Population

Outcomes	Empirical antibiotics			*P*-value
	Non-carbapenem^†^ (N = 26)	Carbapenem (N = 27)	Total (N = 53)	
30-day all-cause mortality	1 (3.8%)	8 (29.6%)	9 (17.0%)	0.01
Microbiological failure on day 2	7 (26.9%)	11 (40.7%)	18 (34.0%)	0.29

^†^In the non-carbapenem group, of in vitro susceptible appropriate empirical antibiotics (n = 6), there were no fatal cases and 3 cases with microbiological failure on day 2. In the non-carbapenem group, of in vitro resistant inappropriate empirical antibiotics (n = 20), there was one fatal case and 4 cases with microbiological failure on day 2

### Impact of initial empirical antibiotics on microbiological and clinical outcomes

In the univariate analysis, statistically significant risk factors associated with microbiological failure on day 2 were being a preterm infant [odds ratio (OR) 42.5; 95% confidence interval (CI) 4.73–381.72], having an intra-abdominal infection (OR 13.1; 95% CI 1.39–122.85), and having a severe clinical presentation (OR 5.0; 95% CI 1.44–17.37) (Table 4). After adjusting for confounding factors including initial empirical use of non-carbapenems and variables with *P*-value < 0.05 in the univariate analysis, only being a preterm infant was associated with 2-day microbiological failure (adjusted OR 30.6; 95% CI 2.43–386.19). However, there was no statistically significant association of use of a non-carbapenem agent as an initial empirical antibiotic with microbiological failure on day 2.

**Table 4:** Factors Associated with Persistent Bacteremia by Day 2 and 30-day All-cause Mortality

Variables^†^	Microbiological failure on day 2		30-day all-cause mortality	
	OR (95% CI)	Adjusted OR^‡^ (95% CI)	OR (95% CI)	Adjusted OR^‡^ (95% CI)
Initial empirical use of non-carbapenems	0.5 (0.17–1.71)	1.0 (0.22–4.88)	0.1 (0.01–0.83)	0.1 (0.01–1.56)
Preterm infant	42.5 (4.73–381.72)	30.6 (2.43–386.19)	15.6 (2.94–82.84)	7.6 (1.04–56.07)
Intra-abdominal infection	13.1 (1.39–122.85)	1.8 (0.08–42.72)	2.9 (0.44–18.68)	NA
Urinary tract infection	0.6 (0.06–6.31)	NA	NA	NA
Initial presentation with clinically severe infection	5.0 (1.44–17.37)	1.2 (0.18–8.03)	11.9 (2.13–66.62)	3.2 (0.40–25.25)

*CI* confidence interval, *NA* not applicable, *OR* odds ratio

^†^Univariate analysis was performed using the variables of initial empirical antibiotics, preterm infants, clinical diagnosis and clinical severity at presentation with *P-*values < 0.1 in Table 1. However, the factor ‘urinary tract infection’ as an infection focus was not included in 30-day all-cause mortality because none of the cases were fatal

^‡^The multivariate analysis was performed using the following variables: empirical use of non-carbapenems and those with *P*-value < 0.05 in the univariate analysis

With respect to the 30-day all-cause mortality in patients with BSIs caused by ESBL-producing *E. coli* and *K. pneumoniae*, being a preterm infant was the only independent risk factor (adjusted OR 7.6; 95% CI 1.04–56.07) after adjustment for receiving initial empirical therapy with non-carbapenem, being a preterm infant, and having a severe clinical presentation.

## Discussion

Our retrospective analysis of pediatric BSIs caused by ESBL-producing *E. coli* and *K. pneumoniae* with a 30-day mortality of 17% suggested that administration of non-carbapenem antibiotics as an initial empirical antibiotic was not a significant risk factor for 30-day mortality or microbiological failure on day 2 if early transition to a definitive carbapenem regimen was possible when susceptibility was proven. Being a preterm infant was the only significant risk factor for 30-day all-cause mortality of BSIs caused by ESBL-producing *E. coli* and *K. pneumoniae* in this study.

Based on the data from our institute from 2009 to 2018, the resistance rates of *E. coli* and *K. pneumoniae* to 3rd and/or 4th generation CSs were as high as 40–50%, and their resistance rates to piperacillin/tazobactam were approximately ≤ 20% (13, 26). If the antimicrobial resistance rate to a certain antibiotic in a hospital or community is higher than 10–20%, clinicians should consider using a different antibiotic or combination therapy as an empirical regimen (27–29). Considering the high ESBL-positive rate of *Enterobacteriaceae* at our institution, if the strain is reported as a Gram-negative strain in the blood culture reporting system, the treatment is first changed to a carbapenem; then, de-escalation is performed if necessary. In cases of septic shock, carbapenems tend to be used as empirical antibiotics. In suspected CNS infections, meropenem is the empirical choice of antibiotic to achieve CNS penetration because the usual doses of tazobactam have been considered to be insufficient for appropriate treatment of CNS infections (30), although few studies have reported the successful treatment of patients with CNS infections with piperacillin/tazobactam (31). So, carbapenems could be easily used as the initial regimen, especially in high-risk patients. However, considering the positive correlation between antibiotic burden and AMR (32–34), administration of broad-spectrum antibiotics should be considered carefully.

Carbapenems have been considered the best choice for definitive therapy in serious infections caused by ESBL producers (35). However, there is controversy regarding appropriate initial empirical antibiotics in both pediatric and adult patients infected with ESBL producers. Inappropriate initial empirical antibiotic treatment has been associated with increased mortality in patients with bacteremia caused by ESBL-producers (36, 37). In contrast, one multicenter study including approximately 10% of pediatric patients aged < 18 years suggested that inappropriate empirical therapy within < 24 h was not associated with increased mortality (38). Our single center study suggested that there is no significant advantage in choosing a carbapenem as an initial antimicrobial agent in terms of the microbiological failure rate by day 2 and 30-day mortality. However, this could not be applied in general. The microbiological laboratory of our institute operates the blood culture system 24 h a day, year-round; when an automatic signal is reported from the BACTEC blood culture system, laboratory personnel perform Gram staining and report the results to an electronic medical record in real time. Therefore, in the case of Gram-negative bacteria isolated from a blood culture, it usually takes less than 24 h to report the automatic positive signal with the Gram-staining result for a corresponding one. Therefore, most patients with BSIs caused by ESBL-producing *Enterobacteriaceae* treated with non-carbapenems as initial empirical antibiotics can be switched to a carbapenem as the definitive therapy within two calendar days, if necessary; furthermore, more than half of them can be switched within one calendar day. These conditions might explain why non-carbapenems as empirical antibiotics were not associated with an increased mortality rate or microbiological failure rate in our study.

Neonates were more likely than older children to develop bacteremia caused by ESBL producers (10), and infections with ESBL producers in NICU were associated with prematurity (39). In this study, 66.7% (6/9) of fatal cases occurred into premature infants with or without NEC.

Considering the high prevalence of bacteremia caused by ESBL-producing *Enterobacteriaceae* and high fatality rates regardless of types of initial empirical antibiotics in neonates (12, 40–43), delicate care and appropriate management for premature infants infected with ESBL producers are necessary to minimize morbidity and mortality.

There are limitations in assessing the causal relationship of some phenomena because this was a retrospective observational study. The more severe the initial symptoms and signs were, the more likely the physician was to administer carbapenems from the beginning, especially for premature infants with septic shock. Most of the fatal cases in this study were associated with premature infants. Even though we performed a multivariate logistic analysis to adjust for possible confounding factors, the sample size is just too small to draw definite conclusions about the effect of initial empirical antibiotic on microbiological failure or mortality while taking into account other risk factors. Furthermore, it was not possible to analyze the statistical difference between the 30-day mortality according to the appropriateness of empirical antibiotics used in the non-carbapenem group. However, no fatal cases were observed among patients who received appropriate empirical non-carbapenem antibiotics. In addition, we did not identify virulent factors, AMR mechanism including specific ESBL genes or clones, and host immune responses, which could be an important way to explain the outcomes of the ESBL producing G(−) bacteremic patients (44), because our study was a retrospective study, and PCR test for ESBL genes was not routinely performed at our institute. Although our result cannot be extrapolated to the general pediatric population, these findings suggest that it is not always necessary to select a carbapenem as an initial empirical antibiotic for ESBL-producing *E. coli* and *K. pneumoniae* bacteremia in children if the appropriate definitive antibiotics can be administered without delay with the support of a culture reporting system. Laboratory-based antimicrobial resistance surveillance systems can incorporate epidemiological data in determining appropriate empirical antibiotics in high endemic setting while balancing between selecting appropriate empirical antibiotics and minimizing antibiotic pressure.

In conclusion, if early transition to carbapenems as a definitive antimicrobial therapy is possible, initial empirical use of non-carbapenems may be considered an appropriate option in pediatric bacteremia caused by ESBL-producing *E. coli* and *K. pneumoniae*. A large multicenter study should be conducted to identify appropriate carbapenem-sparing options for medically fragile pediatric patients and to minimize the spread of MDR pathogens such as carbapenem-resistant bacteria.

## Supplementary Information

**Additional file 1.** Patients data adminitrated with non-carbapenem as empirical treatment.

## Data Availability

All data generated or analysed during this study are included in this published article and its additional information files.
